# Demographic Pattern and Clinical Characteristics of Patients Undergoing Haemodialysis in a Tertiary Centre of a Developing Country: A Review of 280 Cases

**DOI:** 10.4314/ejhs.v33i6.10

**Published:** 2023-11

**Authors:** TA Bamikefa, PK Uduagbamen, MA Adelaja, O Ala

**Affiliations:** 1 Renal Unit UniOsun Teaching Hospital Osogbo, Osun State/ Department of Medicine College of Health Sciences Osun State University Osogbo; 2 Division of Nephrology and Hypertension, Department of Internal Medicine, Bowen University/Bowen University Teaching Hospital, Ogbomosho, Nigeria; 3 Saint Nicholas Hospital 57 Campbell Street Lagos Island, Lagos Nigeria; 4 Endocrine and Metabolism Unit UniOsun Teaching Hospital Osogbo, Osun State/ Department of Medicine College of Health Sciences Osun State University Osogbo

**Keywords:** Haemodialysis, Chronic kidney disease, Renal replacement therapy, Demographic pattern, short-term survival

## Abstract

**Background:**

Globally, renal replacement therapy especially haemodialysis remains pivotal in the effective care of patients with kidney diseases since its acceptance as a treatment modality. Despite being widely embraced as a therapeutic option, several factors still hamper its utilization. A clinical audit of this modality option will allow elucidation of haemodialysis practises and peculiarities.

**Methods:**

The charts and records of 280 patients with renal impairments dialyzed between March 1^st^ 2019 and February 28^th^ 2023 were evaluated in retrospect. Data on retrieved demographic and clinical information were analyzed using SPSS 25 and patients' short-term survival was determined using the Kaplan Meier survival analysis and log rank test.

**Results:**

Out of the 280 patients who had 1716 dialysis sessions, 184 (65.7%) were males. The mean age was 47.9 ± 17.5 years. The majority (80.7%) of the patients had chronic kidney disease (CKD), as 90.2% of the dialysis sessions were for CKD. There was a male preponderance (69.1%) in the population. Hypertension was the commonest cause of CKD (41.2%) while sepsis was the commonest cause of acute kidney injury (50%). The median number of dialysis session was 4.0. The mean pre-dialysis hematocrit was 24.4 ± 7.1% and the mean single pool Kt/V was 0.9 ± 0.02. The femoral vein was the most used vascular access (95.4%). The short-term survival was positively related to the dialysis frequency on Kaplan-Meier analysis.

**Conclusion:**

Haemodialytic therapy in patients with renal disease is still of huge impact on survival despite the numerous factors affecting its effective delivery, especially in low-income nations.

## Introduction

The skyrocketing increase being experienced numerically in the pattern of kidney diseases globally and domestically has continued to generate debate and discussions on the best approach to stem this accelerating tide to avoid a public health disaster now and in the foreseeable future (1). Acute kidney injury (AKI) which is typified by often amenable insults to the kidney in combination with chronic kidney disease (CKD) progressively contributes a huge proportion to the global pool of end stage kidney disease (ESRD) patient dependent on the available but costly and occasionally limited forms of renal replacement alternatives world-wide (2).

Although haemodialysis (HD) still ranks first as the most sought-after renal replacement alternative, its efficiency is still hampered by geographical, economic, and patient-related factors both globally as well as locally (3). The geographically defective utilization of HD in Sub-Saharan Africa compared to the developed countries was brought to the fore in a recent study involving close to 200 countries with an average < 15.0 usage per million population for African countries (4). The lopsided burden of infectious and non-communicable diseases (NCD) causes of renal impairment borne by Sub-Saharan Africa has made the impact of the rising tide of CKD worse in the continent where there are no equitable distribution of HD facilities and often inadequate budgetary plan for health (4-6).

The un-toward consequences of defective utilization of sparse haemodialysis facilities in the developing countries including Nigeria is magnified due to poverty and knowledge deficiency as a lot of patients with renal disease requiring haemodialysis lacks financial capacity to match the ever-increasing demands associated with holistic renal care with resultant high default rate (5-7). This contrasts with documentations from the western world (3,4,8-10). Consequent upon the central role of HD as a penultimate treatment goal to renal transplantation in CKD patients, it has become imperative to identify and understand the pertinent issues involved in its practices to allow for its prompt initiation and optimal prescription.

We highlighted in this retrospective study, spanning 4-years, the peculiarities in the practice of haemodialysis in a tropical tertiary healthcare facility and compared these findings to international best practices and guidelines with a view of improving local practices and proffered solutions to the identified challenges. Knowledge gaps between newer trends and existing practices were identified and attempts made to reduce the widening gap.

## Patients and Methods

**Study design and setting**: A four-year retrospective evaluation of the dialysis chart, clinical notes and records of 280 consecutively recruited patients aged 16 and above who had haemodialytic therapy spanning between March 1^st^ 2019 and February 28^th^ 2023 was undertaken after obtaining due permission of the Ethics and Research Committee of Osun State University Osogbo, Nigeria, with ethical clearance (protocol number UNIOSUNHREC2022/001E). The haemodialysis unit of UniOsun Teaching Hospital, located in Osun State, has continued to render care to patients with acute kidney injury and those with chronic kidney disease since its establishment in 2005. It provides salvage and maintenance haemodialysis services to approximately 2 million people as it is a referral center to primary, secondary and some neighboring tertiary centers with spread to surrounding South-Western states and occasionally North Central states.

**Population**: During the period under review, a total of 280 patients had haemodialysis done and were all included in the study. All these patients were dialyzed using Surdial X machines manufactured by Nipro Corporation Osaka, Japan which utilized bicarbonate-based dialysate. The blood and dialysate flow rate were set at 500mls/min and 200-400mls/min respectively depending on the vascular route and hollow-fibre dialyzer surface area employed.

**Data collection**: Information relating to the demographic attributes and clinical characteristics such as aetiology of renal impairment, type of kidney dysfunction, trigger(s) of acute worsening of background renal injury, referral portal into dialysis unit, length of therapy sessions, summation of dialysis sessions, single pool kt/v, frequency of HD therapy as well as pre-dialytic investigation results were entered into a structured questionnaire for each study respondent. Other information obtained were vascular route for dialysis, pre-and intradialytic blood pressure trends, intra/post dialytic un-toward event(s), use of erythropoietin, need for blood transfusion and clinical outcome.

**Operational definitions**: All clinical diagnosis was done as stated by the attending physician and or nephrologist in the clinical notes of the study respondents as informed by obtained detailed history, examinations, and relevant investigation results.

**Data analysis**: Data extracted were analyzed with Statistical Product and Service Solutions (SPSS) version 25. Charts and tables were used for data presentation as appropriate. Chi-square was employed to compare percentages (categorical variables) while Student T-test was used to compare means (continuous variable). Analysis of Variance (ANOVA) was used to compare means between patients with AKI, CKD and ESRD. Survival trend of the study respondents was determined using Kaplan-Meier analysis and log rank test. Statistical significance was set at a p-value of < 0.05.

## Results

Within the 4-years under evaluation, a total of 280 patients had haemodialysis done out of which one 184 were males (n=184,65.7%) and 96 were females (n=96,34.3%) resulting in a M:F ratio of 1.9:1. The age range of the respondents was between 13 and 96 years with a mean age of 47.9 ± 17.46 years. The mean age of the male participants was higher than that the females (50.6±17.31 years versus (43.0 ±16.72 years). There were significant gender differences in the age-groups (p=0.045), occupation (p=0.007) and marital status (p=0.012) (Table 1).

**Table 1:** Gender distribution of socio-demographic attributes of patients offered HD from March 1^st^, 2019, and February 28^th^ 2023. Nigeria (n=280)

Sociodemographic feature	Male n (%)	Female n (%)	Total n (%)	P-value
Age Group (years)				
11-20	6 (3.3)	11(11.5)	17 (6.1)	0.045*
21-30	19(10.3)	13 (13.5)	32 (11.4)	
31-40	32 (17.4)	22 (22.9)	54 (19.3)	
41-50	38 (20.7)	19 (19.8)	56 (20.0)	
51-60	42 (22.8)	17 (17.7)	59 (21.1)	
61-70	20 (10.9)	9 (9.4)	30 (10.7)	
71-80	18 (9.8)	5 (5.2)	23 (8.2)	
81-90	8 (4.3)	0	8 (2.9)	
91-100	1 (0.5)	0	1 (0.4)	
Total	184 (65.7)	96 (34.3)	280 (100)	
Marital Status				
Single	28 (10.0)	19 (6.8)	47 (16.8)	0.012*
Married	156 (55.7)	73 (26.1)	229 (81.8)	
Widow	0 (0.0)	4 (1.4)	4 (1.4)	
Total	184 (65.7)	96 (34.3)	280 (100)	
Occupation				
Artisan	46 (25.0)	15 (15.6)	61 (40.6)	0.007*
Civil servant	30 (16.3)	11 (11.5)	41 (27.8)	
Trader	54 (29.3)	40 (41.7)	94 (71.0)	
Student	17 (6.1)	14 (5.0)	31 (11.1)	
Retired	21 (11.4)	5 (5.2)	26 (16.6)	
Self/un-employed	7 (3.4)	5 (3.2)	12 (6.6)	
Dependant	9 (4.9)	6 (6.3)	14 (11.2)	
Total	184 (65.7)	96 (34.3)	280 (100)	

* Statistically significant at p-value < 0.05

The majority of the patients were aged between 51 and 60 years (n=42,23%). The peak age in patients with CKD was higher (41-50 years) compared to those with AKI where ages 21-30 and 31-40 had equal frequencies.

The underlying aetiologies of the clinical diagnosis categorized (Table 2) showed a statistically significant relationship between the various causes of AKI across the two genders (p-value=0.010). The most prevalent causes of CKD in the studied population were hypertension (n=93,41.2%), glomerulonephritis (n=51, 22.6%) and combination of hypertension and diabetes mellitus (n=27,11.9%) with male predominance. Sepsis accounted for the majority of the causes of AKI (n=27, 50%) among the respondents with skewness towards the male gender.

**Table 2:** Gender grouping of underlying aetiologies of CKD and AKI in patients offered HD from March 1^st^ 2019 and February 28^th^ 2023. Nigeria (n=280)

Diagnosis	Total n (%)	Male n (%)	Female n (%)	Mean age ± SD years	P-value
CKD	226 (80.7)	156 (69.0)	70 (31.0)	49.2 ±16.4	
Aetiology					
Hypertension	93 (41.2)	70 (44.9)	23 (32.9)	49.7 ± 12.1	0.118
Diabetes Mellitus	11 (4.9)	9 (5.8)	2 (2.9)	61.6 ± 13.0	
Hypertension & Diabetes Mellitus	27 (11.9)	18 (11.5)	9 (12.9)	58.5 ±13.4	
Glomerulonephritis	51 (22.6)	29 (18.6)	22 (31.4)	31.9 ±10.7	
Obstructive Uropathy	18 (8.0)	15 (9.0)	3 (4.3)	57.4 ±16.8	
Hypertension& Obstructive Uro	16 (7.1)	10 (6.4)	6 (8.6)	64.6 ±15.9	
Hypertension, DM & Obstructive	5 (2.2)	3 (1.9)	2 (2.9)	57.6 ±10.7	
Uro					
SLE	2 (0.9)	0	2 (0.9)	40.0 ±12.7	
ESRD	3 (1.3)	2 (1.3)	1 (1.4)	37.6 ±14.6	
AKI	54 (19.3)	28 (51.9)	26 (41.1)	48.8 ±21.0	
Aetiology					
Sepsis	27 (50.0)	14 (51.0)	13 (50.0)	46.8 ± 23.5	0.010*
Hypovolaemia	5 (9.3)	1 (3.6)	4 (15.4)	53.6 ±19.3	
Obstetric	4 (7.4)	0	4 (15.4)	30.0 ± 3.6	
CIN	1 (1.9)	0	1 (3.8)	79.0 ± 0.0	
Acute Glomerulonephritis	2 (3.7)	2 (7.1)	0	20.0 ± 0.0	
Herbicides/herbs	3 (5.6)	3 (10.7)	0	39.7 ±17.0	
Post-operative	6 (11.1)	6 (21.4)	0	40.8 ±14.3	
Multiple Aetiologies	1 (1.9)	1 (3.6)	0	59.0 ± 0.0	
Malignancy	3 (5.6)	0	3 (11.5)	39.7 ± 23.5	
Hb SS	2 (3.7)	1 (3.6)	1 (3.6)	27.0 ± 0.0	

* Statistically significant at p-value of 0.05, DM- Diabetes Mellitus, Obstructive Uro- Obstructive Uropathy, SLE- Systemic Lupus Erythematosus, ESRD- End Stage Renal Disease, CIN- Contrast Induced Nephropathy, HbSS- Haemoglobin SS.

**Figure 1:**
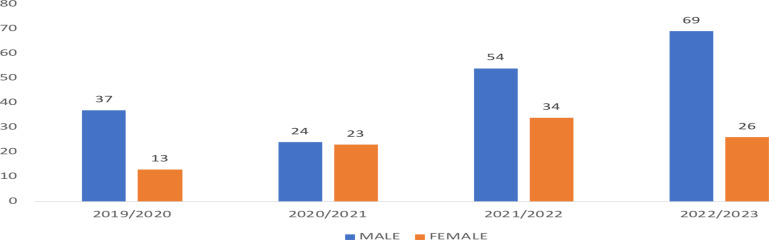
Bar chart showing yearly admission of the study respondents

The highest proportions of male and female respondents were seen in the years 2022/2023 and 2021/2022 respectively (Figure 1). There were significant gender differences in the in-hospital portal of transfer for HD (p-value= < 0.001). More patients came in through the accident and emergency division of the hospital (n=145,51.8%). A minute percentage of the study respondents (n=6, 2.1%) had a prior review by a nephrologist although all the patients had emergency dialysis commencement totaling 1716 sessions with a mean value of 6.1± 6.65 sessions. Chronic kidney disease was responsible for a larger percentage of the cumulative dialysis sessions (n=1547,90.2%) compared to AKI (n=169,9.8%) with therapy sessions ranging between 1 to 50 and 1 to 13 for CKD and AKI, respectively.

Chronic kidney disease contribution to the haemodialysis pool was higher (n=226,80.7%) compared to AKI (n=54, 19.3%). The gender ratio across the clinical diagnosis was 2.2:1 and 1.1:1 for CKD and AKI respectively.

Single pool Kt/v was documented for 211 sessions with a mean of 0.86 ± 0.03. The predominant vascular route for HD was through the femoral veins (n=267, 95.4%) while other vascular access contributed minute percentages (Table 3). Gender contributed significantly to dialysis frequency among CKD patients (p-value= 0.045). The majority of CKD patients had between 4 to 12 sessions of dialysis. The mean pre-dialytic packed cell volume was 24.4±7.10%, with males having significantly higher values than females (p-value= 0.003). Sepsis and accelerated hypertension were the main triggers of acute deterioration in patients with background CKD. The other preliminary attributes of the patients are as depicted (Table 3). There were significant gender differences between triggers of acute decline in kidney function in CKD (p=0.04).

**Table 3:** Gender stratification of preliminary clinical parameters of patients offered HD from March 1^st^, 2019, and February 28^th^ 2023. Nigeria (n=280)

Variables	Total	Male	Female	P-value
Pre-dialysis PCV (%)	24.4 ± 7.10	25.4± 7.21	22.5±6.53	0.003*
SpKt/v	211			
≤0.8	106	70 (51.1%)	36 (48.6%)	
0.9-1.2	85	58 (42.3%)	27 (36.5%)	0.139
>1.2	20	9 (6.6%)	11 (14.9%)	
Dialysis Access	280 (100%)			
Femoral	267 (95.4%)	173 (94%)	94 (97.9%)	
Internal jugular	4 (1.4%)	4 (2.2%)	0	
AV fistula	2 (0.7%)	2 (1.1%)	0	0.501
Tunnelled IJV	5 (1.8%)	3 (1.6%)	2 (2.1%)	
Femoral/Int Jug	1 (0.4%)	1 (0.5%)	0	
Subclavian	1 (0.4%)	1 (0.5%)	0	
Dialysis frequency	280 (100%)	184 (65.7%)	96 (34.3%)	
≤ 3	128 (45.7%)	81 (44.0%)	47 (49.0)	0.045
4-12	131 (46.7%)	84 (45.5%)	47 (49.0)	
> 12	21 (7.5%)	19 (10.3)	2 (2.1)	
Precipitants of AKI in CKD patients	226 (100%)	156 (69.0%)	70 (31.0%)	
Sepsis	86 (38.1%)	59 (37.8%)	26 (37.1%)	
Accelerated HTN	70 (31.0%)	54 (34.6%)	16 (22.9%)	
Nephrotoxins	31 (13.7%)	23 (14.7%)	8 (11.4%)	
Acc HTN& Sepsis	4 (1.8%)	2 (1.3%)	2 (2.9%)	0.041*
Acc HTN& Toxins	11 (4.9%)	7 (4.5%)	4 (5.7%)	
Sepsis & Toxins	4 (1.8%)	0	4 (5.7%)	
Hypotension	1 (0.4%)	1 (0.6%)	0	
Unknown	18 (8.0%)	9 (5.8)	9 (12.9%)	
Nos of Precipitant				
Of AKI in CKD Px				
0	18 (8.0%)	9 (5.8%)	9 (12.9%)	
1	191 (84.5%)	138 (88.55)	53 (75.7%)	0.049*
2	17 (7.5%)	9 (5.8%)	8 (11.4%)	
Nos of Aetiology of CKD				
1	110 (48.6%)	82 (52.6%)	28 (40.0%)	
2	44 (19.5%)	29 (18.6%)	15 (21.4%)	0.202
3	72 (31.9%)	45 (28.8%)	27 (38.6%)	

* Statistically significant at p-value 0f 0.05, Acc HTN- Accelerated hypertension, Nos- Number, AKI- Acute kidney injury, Int Jug-Internal Jugular vein, CKD- Chronic Kidney Disease, Px-patient, SpKt/v- single pool Kt/v, PCV-Packed cell volume

Of the 280 participants, 259 (92.50%), with CKD-202, AKI-55 and ESRD-2, could afford dialysis for a week coupled with delay in securing funds for commencement of therapy. Only 4 (1.4%) participants with CKD could continue HD beyond the 4th week. There were significant differences in the clinical diagnoses relating to the number of dialysis sessions (P<0.001), pre-dialysis serum sodium (p=0.017), pre-dialysis PCV (p=0.001) and pre-dialysis creatinine (p=0.001) (Table 4). Patients with AKI had the highest PCV level compared to other clinical diagnosis (p=0.001).

**Table 4:** Preliminary clinical parameters of patients offered HD from March 1^st^, 2019, and February 28^th^ 2023 categorised based on clinical diagnosis. Nigeria (n=280)

Parameters	CKD	ESRD	AKI	F-value	P-value
Age (yrs)	49.2 ± 16.43	37.7±14.57	43.7±20.69	2.747	0.066
Serum HCO_3_ (mmol/L)	16.9 ± 2.25	16.5 ± 0.71	16.9 ± 1.85	0.038	0.963
Serum Na^2^ (mmol/L)	130.6 ± 5.55	131.5 ± 6.36	133.1± 5.22	4.146	0.017*
Serum K^+^ (mmol/L)	4.8 ± 4.26	4.5 ± 1.48	5.3 ± 5.53	0.254	0.776
Pre-dialytic PCV (%)	23.4 ± 6.40	23.0 ± 1.41	27.7 ± 8.53	7.296	0.001*
Serum Urea (mmol/L)	29.2 ± 9.09	25.5 ± 9.19	27.7 ± 7.99	0.696	0.500
SerumCreatinine (µmol/L)	1279.0 ± 607.5	1201.5± 596.1	873.7± 306.8	9.938	0.001*
SpKt/v	0.86 ± 0.39	1.20 ± 0.00	0.85 ± 0.35	0.796	0.452
Dialysis sessions	6.85 ± 7.13	8.00 ± 11.27	3.20 ± 2.29	7.149	0.001*
SBP Intradialysis (mmHg)	154.23 ± 61.49	146.50 ± 68.59	136.67±50.74	0.692	0.502
DBP Intradialysis (mmHg)	84.94 ± 34.55	84.0 ± 43.84	74.20 ± 27.09	1.233	0.294
Pre-dialytic SBP (mmHg)	158.42 ± 28.09	177.0± 5.66	155.93 ±22.64	0.597	0.552
Pre-dialytic DBP (mmHg)	91.44 ± 17.50	108.50 ±12.02	89.33 ± 15.63	1.214	0.300
Frequency of dialysis n (%)					
1^st^ Week	202 (91.1)	2 (66.7)	55 (98.2)		
2^nd^ Week	9 (44.1)	7 (33.3)	1 (1.8)	10.381^#^	0.109
3^rd^ Week	6 (2.7)	0	0		
4^th^ Week	4 (1.8)	0	0		

* Statistically significant at P-value of 0.05

# X-value, PCV- Packed cell volume, SpKt/v- Single pool Kt/v, SBP- Systolic Blood pressure, DBP- Diastolic Blood pressure.

Chronic kidney disease patients had a higher occurrence of hyperkalaemia (38.1% versus 29.6%) and anaemia (62.8% versus 51.9%) compared with AKI although acidosis was more prevalent amongst patients with AKI (72.2% versus 70.4%). On multivariate regression analysis, all the statistically significant clinico-demographic parameters did not exhibit any predictive tendencies. Out of the 280 patients offered HD therapy, 256 (91.4%) were discharged (CKD=206, AKI=50) while 14 (5%) died. Only 2 (66.6%) of the ESRD cases could continue with twice weekly sessions. Intradialytic hypotension (IDH) was more common than intradialytic hypertension (IDHT) (20.7% vs 12.9%).

**Figure 2:**
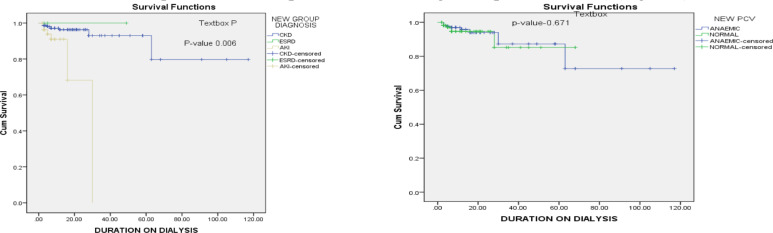
Kaplan Meier Survival plots on HD patient's survival taking into cognisance length of HD therapy, type of kidney disease and presence/absence of anaemia

All-cause mortality for AKI and CKD were 11.1% and 3.5% respectively. Kaplan Meier survival analysis revealed a statistically significant effect of duration of dialysis on the cumulative survival of patients on HD (p-value=0.006) with improved outcome in patients with AKI compared to CKD/ESRD (Figure 2). Anaemia (PCV < 30%) did not contribute significantly to survival across the clinical diagnosis (p-value= 0.671) (Figure 2).

## Discussion

The study was undertaken to elucidate the clinico-demographic peculiarities of 280 patients with varying degrees of renal dysfunction who had HD within the 4 years under review. Our study showed a heightened socio-economic burden of renal impairment particularly that requiring HD on disease victims compared to findings from two previous studies conducted in the same facility (7-8). These exacerbations have continued nation-wide despite the emergence of more renal replacement facilities though unevenly distributed throughout the country with no effective government intervention (s) to ameliorate the sufferings of these group of patients who cannot initiate and or sustain HD due to financial handicaps (5-6,11).

In North-Eastern part of Nigeria, a total of 226 patients underwent HD over a 7-year period compared to this study where 280 patients had HD over a 4-year period further giving credence to the rising burden of renal diseases locally as well as globally occasioned by the interplay between various factors underlying NCD accentuation (4-6,12). The male preponderance in our study population is in agreement with previous findings, largely attributable to socioeconomic and cultural biases against women in our clime as well as lopsided referral practices (13-16). The apoptotic effect of testosterone on the renal tubules coupled with estrogens' renal protective actions may also be contributory (17-18).

The higher mean age of males mirrors previous findings in Nigeria and Ethiopia (7-8,10). This is similar to previous findings in this facility and other Southern states although higher compared with observations in the Northern part of the country (7,8,12,13,14). The mean age of patients with ESRD was lower compared to CKD and AKI patients giving further credence to the effect of foreign diets, greater stress involved in attaining higher standards of living resulting in elevated blood pressures, and a greater use of nephrotoxins (creams, soaps and eye lashes/creams) in the development and progression of CKD in the young. Lower ages of patients with renal dysfunction have also been documented in other climes compared with the western-world (9-10,20-21). The worrisome trend of increased occurrence of kidney diseases among the productive young workforce in the developing countries compared to the developed nations, where it majorly affects the older age groups, calls for pro-active and effective measures to stem this unfavorable tide (7-10,20-21).

Presentation for dialysis in an acute and often time life-threatening states as demonstrated in this study could largely be attributed to socioeconomic deprivation and poor knowledge of kidney disease and its common risk factors such as hypertension, diabetes and dyslipidaemia. The distance between some communities and dialysis facilities, dwindling renal care experts, coupled with bad road network, could only worsens this pattern. These factors could also contribute to the higher prevalence of temporal dialysis vascular access, particularly femoral in our center for dialysis commencement as they are cheaper and require less expertise in their placement as opposed to artero-venous fistula (AVF) creation (4,8-12,20).

The elucidation of CKD remotely followed by AKI as the predominant conditions necessitating HD initiation has been reported in previous local and foreign studies. The predominant CKD-aetiologic factors in the study population were closely comparable to previous findings in this facility and other tropical countries with significant contributions from hypertension, glomerulonephritis and combination of DM and hypertension (5,7-8). The predominance of hypertension as the commonest aetiology of CKD may be due to evolving dietary practices in Africans with a tilt towards western diets, improved spending capacity, urban lifestyle as well as genetics (5,22). Glomerulonephritis and DM in varying proportions has been reported as the major cause of CKD in some parts of Nigeria as well as Ghana and Ethiopia (9-10,17-18).

Sepsis predominated as the most frequent cause of AKI as previously reported although in varying proportions further buttressing the need for prompt and effective infection control to reduce the occurrence of AKI which is a risk factor for future development of CKD (5,7-8,23-24). The most frequently encountered cause of acute worsening of renal function in patients with pre-existing CKD was sepsis closely followed by accelerated hypertension and exposure to renal toxins. These triggers can be reduced with proper patients' education on the need to abstain from indiscriminate use of over-the-counter medications, herbal preparations and to take their antihypertensives as at when due in addition to effective government legislation and health insurance. This is to mitigate the adverse consequences of these precipitants especially hypertension and cardiovascular dysfunction, common associates of cardiac events and sudden death among HD patients (25,26).

The higher pre-dialytic potassium level observed in patients with AKI compared to CKD and ESRD could be contributory to the higher all-cause mortality among those with AKI through its cardiac hyper-excitatory properties (27). The higher pre-dialytic BP and serum creatinine in CKD and ESRD compared to AKI may be attributed to often common cardiovascular comorbidities synergizing with the advanced renal dysfunction in CKD and ESRD, further mitigating against effective blood pressure control in CKD and ESRD. These findings mirror the observation by Prakash et al in Hong Kong (26). Though pre-dialysis haematocrit levels were higher in AKI (27.7± 8.53%) compared to CKD (23.4± 6.40%) and ESRD (23.0 ±1.41); it was still below the recommended value of 33-36%, and this pattern has been widely reported globally (2-4,7-12, 28). Although anaemia has a significant negative impact on morbidity and mortality in renal dysfunction, it remained a particularly difficult challenge in this environment due to the very high cost of erythropoietin (EPO), which has necessitated higher blood transfusion rates with attendant consequences. In this study, only those with ESRD had EPO albeit irregularly.

The prevalent pre-dialytic hyponatremia observed in this study could have resulted from the common prescription of low salt intake in association with diuretics therapy, a common and essential antihypertensive regimen in blacks. Unfortunately, this has been associated with a poorer outcome and with diverse intradialytic complications (29,30). Dialysis adequacy among all the HD recipients using single pool Kt/V was within normal range in < 10%. Higher value gotten for patients on maintenance HD may be attributed to the utilization of jugular access which can accommodate higher dialysate and blood flow rate compared to femoral access (31).

The occurrence of wide blood pressure fluctuations as with IDH (20.7%) and IDHT (12.9%) in this study has been widely reported and may be due to a combination of factors such as the underlying aetiology of renal dysfunction, advancing age, and malnutrition, among other factors (5-12,32). The all-cause mortality of 5% in this study, though lower than that from a previously reported finding from this facility, may be falsely so as the majority of the patients discharged were lost to follow-up while some opted out of treatment on account of financial constraints, as there is no governmental intervention in dialysis care in our environment (8).

The retrospective nature of the study was a major limitation as proper recording and chart-keeping could be poor. The relatively small sample size and single center design coupled with lack of a national central renal registry may make inferential statistics very challenging. Lack of governmental and private sector involvement in renal care, coupled with widespread poverty in the general population, also limited the panel of useful investigations that were conducted.

In conclusion, the demographic pattern and clinical attributes of patients who had HD in our facility were not at variance with previous findings in our clime. Hypertension and infections, being major culprits in the aetiology of both acute and chronic kidney disease, require more concerted efforts and widespread collaborations to mitigate their occurrence and effects. This should involve the government at all levels, multinational corporations, philanthropists and religious bodies to strengthen effective preventive measures from primary to tertiary levels to minimize the occurrence and progression of kidney disease. The government should also enact laws that will support all aspects of renal replacement care coupled with provision of effective health insurance as well as implementation of tax waiver on dialysis consumables.
